# Bayesian Fusion Model Enhanced Codfish Classification Using Near Infrared and Raman Spectrum

**DOI:** 10.3390/foods11244100

**Published:** 2022-12-19

**Authors:** Yi Xu, Anastasios Koidis, Xingguo Tian, Sai Xu, Xiaoyan Xu, Xiaoqun Wei, Aimin Jiang, Hongtao Lei

**Affiliations:** 1 Guangdong Provincial Key Laboratory of Food Quality and Safety/Nation-Local Joint Engineering Research Center for Precision Machining and Safety of Livestock and Poultry Products, College of Food Science, South China Agricultural University, Guangzhou 510642, China; 2 College of Light Industry and Engineering, Sichuan Technology & Business College, Chengdu 611800, China; 3 Guangdong Laboratory for Lingnan Modern Agriculture, Guangzhou 510642, China; 4 Institute for Global Food Security, Queen’s University Belfast, 19 Chlorine Gardens, Belfast BT9 5DJ, UK; 5 Public Monitoring Center of Agricultural Products, Guangdong Academy of Agricultural Sciences, Guangzhou 510642, China

**Keywords:** codfish, authenticity, Raman spectrum, near infrared spectrum, Bayes information fusion

## Abstract

In this study, a Bayesian-based decision fusion technique was developed for the first time to quickly and non-destructively identify codfish using near infrared (NIRS) and Raman spectroscopy (RS). NIRS and RS spectra from 320 codfish samples were collected, and separate partial least squares discriminant analysis (PLS-DA) models were developed to establish the relationship between the raw data and cod identity for each spectral technique. Three decision fusion methods: decision fusion, data layer or feature layer, were tested and compared. The decision fusion model based on the Bayesian algorithm (NIRS-RS-B) was developed on the optimal discrimination features of NIRS and RS data (NIRS-RS) extracted by the PLS-DA method whereas the other fusion models followed conventional, non-Bayesian approaches. The Bayesian model showed enhanced classification metrics (92% sensitivity, 98% specificity, 98% accuracy) that were significantly superior to those demonstrated by any of other two spectroscopic methods (NIRS, RS) and the two data fusion methods (data layer fused, NIRS-RS-D, or feature layer fused, NIRS-RS-F). This novel proposed approach can provide an alternative classification for codfish and potentially other food speciation cases.

## 1. Introduction

Seafood is rich in protein and is popular among consumers for its high nutritional value and delicious taste. Meanwhile, seafood is one of the foods most vulnerable to adulteration mainly due to the significant alterations of the species morphological characteristics that occur during the different types of processing, which render the visual species impossible [1]. Consumers’ demands of certain fish (e.g., cod over pollock) increased the potential of seafood fraud such as species substitution, adulteration, origin confusion and mislabeling [2,3]. Therefore, several efforts, including new regulations, have been introduced in the last decades by different countries and organizations around the world to combat seafood fraud [4].

Cod or codfish is a commercially important species of seafood worldwide. The cod usually refers to fish of the family *Gadidae* and to related species within the *Gadiformes* order [5]. It is reported that cod species with higher value are often replaced by other species with lower price [6]. For example, a study in the UK and Ireland tested 226 cod products from various commercial retailers, and found 28.4% of Irish and 7.4% of UK samples to be mislabeled [7]. Indication of origin is very important in the fish sector because its declaration is in many countries mandatory by law. With seafood speciation and raised awareness regarding origin, the development of cod identification technology is of great significance to protect the interests of consumers, improve the risk control measures of import ports and respond to public concerns.

Analytical technology has become a key element of fish identification, with an increasing number of tools developed to detect or reduce the existence of fraud in global seafood supply chains. Sensory, microbial, physical and instrumental methods have been evaluated for identity assessment of seafood [8,9,10]. DNA testing is the most suitable method for authenticity testing, and many DNA-based methods have been developed to detect fish species [1,11]. However, these methods are clearly time-consuming, destructive, unable to achieve rapid detection on site or require trained personnel [12]. In parallel, several spectroscopic techniques combined with chemometrics have been employed [13,14]. These studies have demonstrated the potential of vibration spectroscopy for rapid and non-destructive identity assessment on seafood.

The application of near infrared and Raman spectroscopy in the field of seafood has attracted more and more attention. For example, the ability of visible/near-infrared (VIS/NIR) spectroscopy was evaluated to predict the cold storage time of salmon meat and skin, and a double-layer stacked denoising autoencoder neural network (SDAE-NN) algorithm was introduced to establish the prediction model. The determination coefficient of test sets (R^2^ test) and root mean square error of test sets (RMSEP) have been calculated based on SDAE-NN; for the salmon meat (skin), the R^2^ test can reach 0.98 (0.92), and the RMSEP can reach 0.93 (1.75), respectively. [15]. In addition, Raman spectroscopy was applied using a 532 nm laser for the classification of 12 frozen types of frozen fish fillets. Hierarchical cluster analysis of their spectra showed that groups could be identified. The accuracy of the spectral classification on the species level as shown in the dendrogram was high, at 95.8% [16].

Both Raman and near infrared spectroscopy are fast and non-destructive food identification and detection techniques [17,18]. However, spectral and spatial interpretation remains challenging for the identification of seafood origin using single spectral techniques. This is mainly due to the low sensitivity of near infrared spectroscopy and Raman scattering intensity, which are easily affected by optical system parameters and other factors. To remedy these disadvantages, spectral data fusion technology using the complementary relationship between the single spectral in qualitative detection of molecular groups can also be explored. Data fusion can be carried out at three levels: data layer fusion, feature layer fusion and decision layer fusion. Data and feature layer fusion have been widely used in traceability and quality identification of aquatic products [19]. However, decision level fusion calculates a separate model from each data source and combines the results of each separate model to obtain the final decision. The decision level fusion can complement the results of each of the other spectral methods and make the detection result more comprehensive and accurate [20]. In addition, there is a lack of research on simultaneous identification of cod species and origin. 

Partial least squares discriminant analysis (PLS-DA) is an established regression-based algorithm coupled with discriminant analysis to allow classification. The regression results of PLS are essentially transformed into a set of intermediate linear potential variables that can be used to predict dependent variables. The dependent variable is the given class label, which is used to indicate whether a given sample belongs to a given class. The model based on the above principles can be used to predict the class of new samples [21]. This is the first work to propose a method based on Raman and NIR PLS-DA features combined with a Bayesian decision fusion model to perform the rapid identification and simultaneous analysis of codfish species and geographical origin.

## 2. Materials and Methods

### 2.1. Codfish Samples Preparation

The codfish samples (all belonging to the *Gadiformes*, *Gadidae* family) used in this study originate from major producing countries in the world. The codfish samples were collected from the direct purchases of aquatic product import and export enterprises, which had a high reputation and were registered by the Chinese customs, to ensure the authenticity of the source of samples. All cod caught offshore were treated with ship-frozen preservation, deboned, and cut by high-pressure waterlines, and transported to the end user, maintaining the cold chain to ensure consistency of freshness.

**Figure 1:**
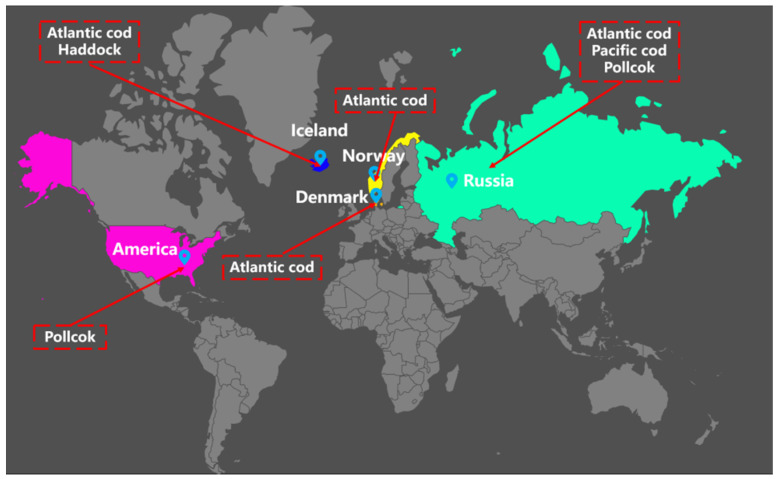
The distribution of collected cod samples in main export countries.

**Figure 2:**
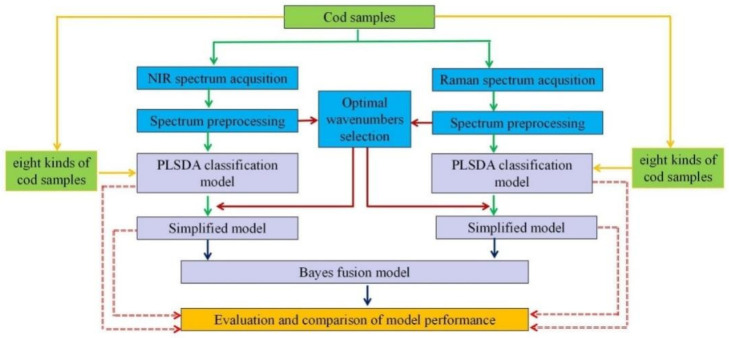
Schematic diagram illustrating the workflow of data processing. Mainly includes spectral data acquisition, preprocessing, feature wavebands selection, partial least squares discriminant analysis (PLS-DA) model construction, Bayesian information fusion and model evaluation.

The samples were mainly from the main cod exporting countries (Figure 1) and processed from belly meat segmented into different-identity codfish. A total of 320 samples (five codfish for each identity, total 40 codfish, all samples divided into 30 mm × 30 mm × 10 mm) were collected, of which 40 samples were from each identity, including eight kinds of codfish with different identities: Atlantic cod from Denmark (ACD), Atlantic cod from Iceland (ACI), Atlantic cod from Norway (ACN), Atlantic cod from Russia (ACR), Haddock cod from Iceland (HDI), Pacific cod from Russia (PCR), Pollock from Russia (PR) and Pollock from America (PA), respectively. All codfish samples were collected from a local fishing company in each region and stored in a −18 °C refrigerator. The key steps illustrating the experimental procedure are summarized in Figure 2 and explained in detail in the following sections.

### 2.2. Spectrometer and Spectral Data Acquisition

#### 2.2.1. Near Infrared Spectrometer

Codfish samples were put into thermal insulation bags with ice cubes and quickly taken to the laboratory. A laboratory-based near infrared spectrometer was used to obtain NIRS of codfish samples in reflectance mode. The treated homogenised cod tissue sample was placed in an aluminum cup, and spectral data were collected by the probe. The near infrared spectroscopy system (Vertex 70, Bruker, Germany) consisted of a nexus optical platform, high-resolution NIRS optical system, interferometer and 24 bits DigiTectTM detector. The data were recorded at room temperature (25 ± 1 °C) in the wavenumber range of 4000–11,000 cm^−1^ at a 1.93 cm^−1^ interval at 8 cm^−1^ resolution, 64 scans. The Fourier transform was automatically applied to the signal to transform the time-based series to frequency. A calibration procedure was performed on the instrument before each sample NIR spectrum collection. Furthermore, each of the samples was measured with the spectrometer three times using a linear movement to obtain mean spectra.

#### 2.2.2. Raman Spectrometer

The RS of samples were recorded in the range from 250 to 2500 cm^−1^ with a Raman setup (laser microscopy confocal Raman spectrometer, Nikon ECLIPSE Ti-U, Tokyo, Japan). Raman scattering was excited by a frequency-doubled Nd/YAG laser at a wavelength of 532 nm with a laser power of about 2 mW incident on the sample. The dispersive spectrometer has an entrance slit of 50 μm and a focal length of 800 mm. The Raman-scattered light was detected by a high-performance charged couple device (CCD) camera. The acquisition time per spectrum was 10 s. To compensate for the use of a microscopy-based instrument instead of standard point-and-shoot to acquire the Raman spectrum, a uniformly homogenised cod tissue sample was produced and measured at three different points.

For the calibration of the Raman spectrometer, optical path collimation was used. This was achieved by ensuring that measured wavelengths were consistent with the values of standard spectral lines. Every day before the Raman measurements, the confocal system was calibrated using a silicon plate (520.7 cm^−1^) provided by the instrument manufacturer to ensure the accuracy of Raman displacement. Then, RS from codfish samples were acquired at a steady level room temperature and humidity. Furthermore, each of the samples was measured with the spectrometer three times to obtain mean spectra.

### 2.3. Data Processing and Multivariate Analysis

#### 2.3.1. Spectrum Preprocessing

All collected spectral data were converted and exported as comma-separated value (CSV) files. For NIRS data, due to the image noise level at the beginning and end of the acquired spectral wavelength bands, the spectral information of a total of 2593 wavenumbers from 4000 to 9000 cm^−1^ was selected for subsequent analysis. The spectral information of a sample collected by a near infrared spectrometer is usually affected by background information and noise interference, and these factors can affect the accuracy of the data analysis. Different pretreatment methods are used to remove or reduce noise and enhance spectral features, which is convenient for more efficient data mining of spectral data. In this research, seven preprocessing algorithms, including normalization (NOR) (model: range normalization), mean centering (MC), multiplicative scatter correction (MSC), standard normal variation (SNV), first derivative (FD), baseline correction (BA), and SNV with MC were employed to preprocess the NIRS [22,23,24,25]. Similarly, for RS data, the spectrum information of a total of 669 wavenumbers from 1000 to 2000 cm^−1^ was selected for subsequent analysis. Seven methods including NOR (model: range normalization), Savitzky-Golay smoothing (SG) (model: smoothing point: 15, order:2, derivative: 0), SNV, BA, SNV with NOR, BA with NOR, SG with NOR were used to preprocess the RS. All the average spectral data were saved in a matrix form (320 × 2593 or 320 × 669) for chemometric analysis, of which 320 rows represented the 320 samples, and 2593/669 columns represented the 2593/669 wavenumbers. The spectrum preprocessing methods were implemented using Unscrambler X 10.4 (CAMO Software AS, Oslo, Norway).

#### 2.3.2. Selection of Important Wavenumbers

In this investigation, a vast array of NIRS or RS data were generated. The average interval of wavenumbers in NIRS and shift in RS were 1.93 cm^−1^ and 1.49 cm^−1^, respectively. Hence, it was necessary to select optimal wavelengths to simplify and improve the predictive models [26,27]. In the current work, three variable selection methods were employed to extract feature wavebands. Iteratively retaining informative variables (IRIV) uses random combinations of variables to take into account the interactions between variables; only the strong information variables and weak information variables are retained, and the analysis of several iterations is carried out at the same time until the remaining variables have no information variables and interference variables [28]. Competitive adaptive reweighted sampling (CARS) obtains variables based on the principle of “survival of the fittest”, and extracts feature wavenumbers after repeated cyclic Monte Carlo sampling [29]. Successive projections algorithm (SPA) selects feature variables with minimal redundancy to solve the collinearity problems. In the SPA process, a projection operation in a vector space is applied to select subsets of variables with a minimum collinearity [30]. The IRIV, CARS and SPA algorithms were implemented in Matlab 2020a (MathWorks Inc., Natick, MA, USA).

#### 2.3.3. Development of Classification Models

To avoid bias in selecting the subset and estimating the performance of a developed model, the calibration and prediction set were comprised of 75% and 25% of the total samples, respectively. The sample split was random, making sure that both sets of data included at least some samples of each subgroup. Partial least squares discriminant analysis (PLS-DA) [31,32] as a supervised linear machine learning technique was utilized to classify the codfish identity. All the samples were divided by the random-grouping method into the training set of 240 samples and the prediction set of 80 samples. The prediction of codfish identity accuracy was performed by applying PLS-DA models based on two different spectral profiles (NIRS, RS) in the full or feature wavebands range.

In the PLS-DA model, the sample category is represented by a binary code group. Each bit is called a node, and each node is represented by “1” as belonging to this class, and “0” as belonging to other classes. There are eight kinds of codfish in this study, so class variables can be represented by eight nodes in the process of model building. PLS regression was performed on each node of all samples to obtain the predicted value of each node. The model obtained searched for directions with the maximum separation among categories, improving the class separability.

#### 2.3.4. Bayes Information Fusion Method

The Bayesian method fully integrates historical prior information and current sample information to carry out statistical inference and parameter estimation [33,34]. The Bayes formula in probability theory is applied to realize the re-decision of NIRS discriminant and RS discriminant. Taking the identification of codfish as an example, the system’s possible decision is *A*_1_~*A*_8_. Two kinds of spectroscopic methods are used to distinguish codfish; the discriminant condition of the NIRS method is B, and the discriminant condition of the Raman spectroscopic method is C. Since A, B and C are independent of each other, the prior probability *P*(*A_i_*) of codfish belonging to all kinds of *A_i_* is equal. In the information fusion method based on PLS-DA, the values of all nodes of each sample of PLS-DA are taken as the probability that the sample belongs to each category, and the probability is input into the Bayesian discriminant formula as the prior probability value. In this process, the information of all nodes of PLS-DA is retained, which is one of the reasons that information fusion contributes to the improvement of the discrimination effect of traceability model in the subsequent result analysis. Here, the posterior conditional probabilities of all kinds of decision *A_i_* (*i* = 1–8) can be expressed as:

(1) P(Ai | B^C)=P(B | Ai)P(C | Ai)∑k=18P(B | Ak)P(C | Ak)

By default, the value of the PLS-DA node is a regression value that may appear to be less than 0 or greater than 1, which is obviously not the range of probability values. To tackle this, we followed a probability-based approach: we set the probability that the node value is less than 0 as 0 and calculated the relative probability of other node values to ensure that the sum of the probabilities of cod identity discrimination is 1. The processed node values were substituted into the Bayesian formula to calculate the a posteriori probability. After the posterior probability was obtained through Bayesian information fusion, the classification of cod samples was judged according to the following two criteria: (1) the target category has the maximum posterior probability, (2) the difference between the target category and other categories must be greater than a certain threshold; in this case, 0.01.

### 2.4. Model Performance Evaluation

To evaluate the performance of the models, the parameters including sensitivity (the true positive results as a fraction of the true positives plus false negatives), specificity (the true negative results as a fraction of false positives plus true negatives) and accuracy (true positives plus true negatives divided by total sample) of calibration (SEC, SPC, ACC), cross validation (SECV, SPCV, ACCV) and prediction (SEP, SPP, ACP) were calculated [35]. The optimal model was developed considering the specificity, sensitivity and class accuracy led to a maximum. The PLS-DA and model performance evaluation were carried out in PLS-TOOLBOX Solo 8.7 (Eigenvector Research Inc., Wenatchee, WA, USA). The Bayesian fusion algorithm was carried out in Microsoft Excel 2010.

## 3. Results and Discussion

### 3.1. Analysis of NIRS Modeling Results

#### 3.1.1. Analysis of the NIRS Features

**Figure 3:**
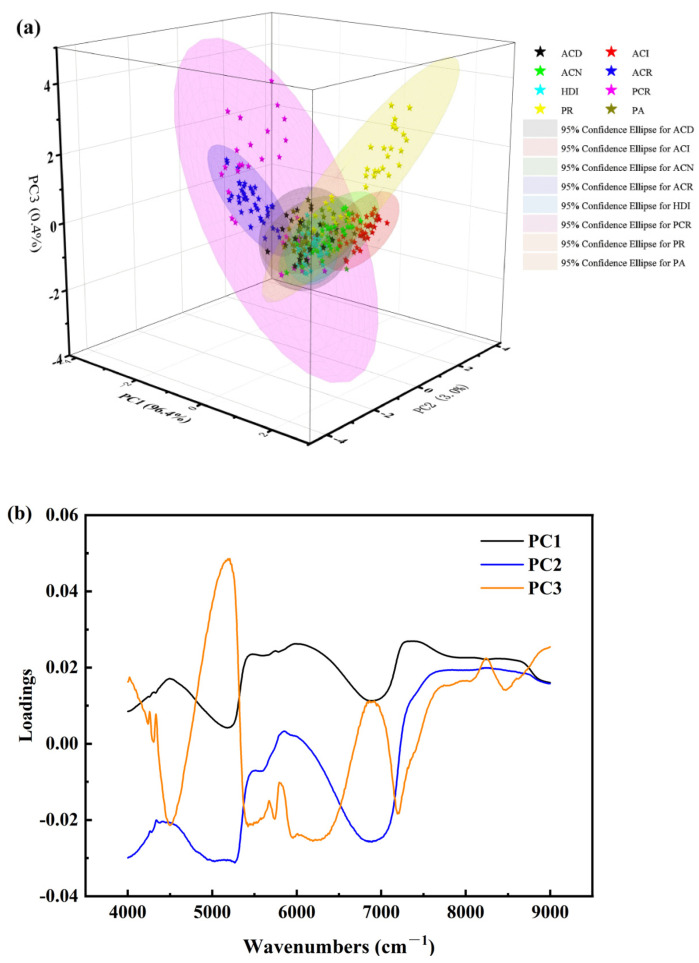
PCA results. (**a**) 3D view of the PCA scores of codfish NIR spectra colored by origins, (**b**) Principal component (PC) loadings.

Similar morphological features of codfish samples were found within the acquired wavenumber region; note that the magnitude of spectra absorbance fluctuates with the identity difference of the cod. NIRS is mainly generated from molecular vibration transition from ground state to a high energy level caused by anharmonicity of the molecular vibration, which contains the chemical bonding information for organic compounds [36]. The peaks and valleys in the NIRS region are mainly caused by the frequency doubling and combined absorption of stretching and bending vibration of hydrogen-containing groups. Figure 3a implied that the differences in identity have induced significant alterations to the samples in a way that can be detected by spectral information. Among them, the absorbance of the peaks and valleys in the three bands of 4000–6000 cm^−1^, 6500–7000 cm^−1^ and 8000–8500 cm^−1^ are more significant than those in other bands, which indicates that NIRS analysis can be used to classify eight kinds of codfish. The Principal Component Analysis (PCA) loading plot also confirmed this (Figure 3a). Most variation in the spectral data was described by the first three principal components. PC1 (96.4% of captured variance) is the main direction along which the samples separated (Figure 3b). It should be noted that the eight types of codfish cannot be well distinguished by PCA unsupervised learning alone.

**Figure 4:**
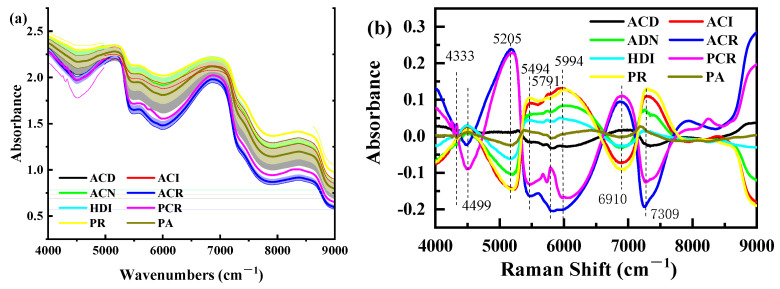
Mean spectra curves of codfish samples. (**a**) NIRS original spectrum, (**b**) NIRS pretreated by standard normal variation with mean centering (NIRS-SNV-MC). Note: NIRS, near infrared spectrum. The mean spectrum and corresponding standard deviation of each measurement group are displayed in different colors, and the standard deviation is indicated by the shading accompanying each mean spectrum line.

The overtones of different molecule bonds following NIRS exposure absorb at specific frequencies that are characteristic of their structure. The NIRS of the cod samples (Figure 4a), showed the first and second overtone of the OH stretching vibrations (6920 and 5145 cm^−1^) due to water. The first and second double frequency of C-H in the region 5555–6250 cm^−1^, 7140–9000 cm^−1^, and the first double frequency of N-H in the region 6250–7140 cm^−1^ can be attributed to protein. At 8387 cm^−1^, there is an absorption band connected to the second overtone stretched by the C-H aliphatic group, which is attributed to fat. SNV with transformations of the spectra (Figure 4b) highlighted further peaks at 4500, 5994, 7309 cm^−1^ originated from protein fraction absorption, i.e., N-H first and second overtone and the combination of N-H and C=O signal [37]. There are minute differences between samples (Figure 3a contains overlays of all NIRS for the 320 samples); therefore, multivariable analysis and chemometrics can be considered to solve the invisible differences of human eyes.

#### 3.1.2. Selection of Pretreatment Methods for NIRS

To eliminate the influence of noise and minimize the miscellaneous scattering, seven standard signal processing methods were employed to pretreat cod original NIRS. The seven preprocessed spectral data were taken as the input of PLS-DA classification model, and the performance of the classification model is shown in Table 1. PLS-DA calibration models were built to correlate the corrected data across full wavelengths with codfish labels, of which the calibration model based on SNV with MC preprocessing yielded acceptable results, with a SEC of 89.81%, SPC of 92.19%, ACC of 89.64% for the calibration set, and SEP of 89.53%, SPP of 90.84%, and ACP of 87.95% for the prediction set. From the results, we can also see that the effect of FD preprocessing is not as prominent as the original spectral modeling. FD and baseline methods amplify the noise in the spectrum, which can explain the poor performance of the models. Hence, SNV with MC was selected as the optimal pretreatment method. Generally, the results were improved by the SNV with MC preprocessing of raw spectra, which may be because the SNV pretreatment reduced the multiplicative effect of scattering [38]; meanwhile, MC pretreatment corrected the relative baseline shift and shift phenomenon between cod samples [39]. Figure 4b demonstrates the differences in NIRS of cod with different identities after pretreatment.

#### 3.1.3. Extraction of Effective Wavenumbers

**Figure 5:**
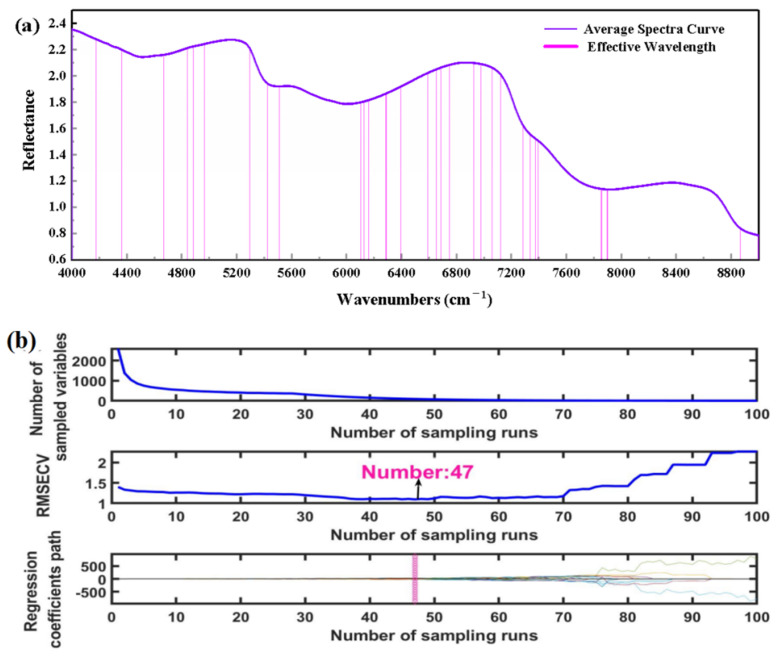
Selection of feature wavenumbers based on (**a**) IRIV-NIR, (**b**) CARS-NIR.

Selection of important wavelengths and minimization of the number of wavebands are very advantageous for building a more stable and comprehensive calibration model. Figure 3b demonstrates that different kinds of cod samples have spectra with overlapping areas that will affect model classification efficiency, so there is a need to reduce spectral data dimensions. Eighty-three wavenumbers (predominately located in 4179–4401, 4671, 4794, 4841–5057, 5157, 5296–5512, 5743, 6106–6981, 7020–7398, 7853–7903, 8067, 8086, 8868–8999 cm^−1^) were obtained by IRIV method for the prediction of codfish identity (Figure 5a). Figure 5b presents the running process of the selection of feature wavenumbers by CARS algorithm, setting it to run 100 times. Figure 5b (the top figure) shows the process of screening the number of characteristic variables, which is divided into two parts; the first stage is rapid reduction (rough selection) and the second stage is very slow (selection). Figure 5b (the middle figure) shows the variation trend of RMSECV. When the minimum RMSECV value is 1.0951, ninety-three characteristic wavenumbers (4000, 4050, 4162–4499, 4557,4559, 4615–4970, 5059, 5138–5518, 5604, 5606, 5984, 5990, 6285–6762, 6924–7060, 7141, 7851–7923, and 8218–8297 cm^−1^) are selected, accounting for 3.58% of 2593 total wavenumbers. Each line in Figure 5b (the bottom figure) represents the changing trend of the regression coefficient, and * indicates the position with the smallest RMSECV. In addition, nine characteristic wavebands (4000, 4353, 4661, 5340, 6611, 7059, 7126, 7759, 8447 cm^−1^) are extracted by SPA algorithm.

#### 3.1.4. Modeling Based on Selected Optimal Wavenumbers

The selected feature wavenumbers were assessed and compared to verify the validity of the selected wavelengths in rapid determination of codfish identity. Table 2 shows the PLS-DA model established by selecting feature wavenumbers by IRIV, CARS and SPA algorithms. As shown in Table 2, although the number of wavenumbers was greatly decreased by the SPA method, the spectral data in the calibration set were reduced to small matrixes as 240 × 9 (samples × variables); the model was showing some over-fitness. This might be because the SPA algorithm lost some useful information related to codfish identity during the extraction of the important wavebands and thus the robustness of the model was reduced. The relatively more accurate model for predicting codfish identity was established using the PLS-DA model based on key variables extracted by the IRIV method; the SECV and SPCV were close to the SEC and SPC of the calibration model, and the ACC value was higher than 90.00% (SEC =98.34%, SECV = 91.26%, SPC = 97.96%, SPCV = 96.3%, ACC = 98.15%, ACCV = 93.78%). These results indicated that the key wavebands identified by IRIV were informative and relevant to the identity of codfish. The number of variables was significantly reduced (by 96.8%) by IRIV, indicating further that the IRIV algorithm was effective in eliminating the redundant information. To further verify the credibility of the simplified IRIV-PLS-DA model, the measured and predicted values of the 80 samples in the prediction set were compared, and the SEP, SPP, ACP were obtained, with 85.00%, 96.25%, 90.63%, respectively. Therefore, it was feasible to use feature wavelengths selected by the IRIV algorithm to represent the original NIRS data (83 vs. 2593) for building the evaluation model of the codfish identity.

### 3.2. Analysis of RS Modeling Results

#### 3.2.1. Analysis of the Spectral Features

**Figure 6:**
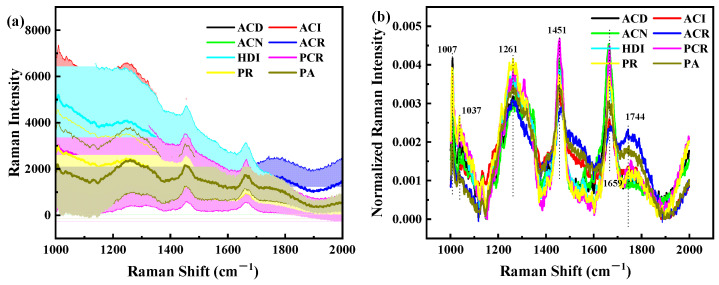
Mean spectra curves of codfish samples. (**a**) Raman original spectrum, (**b**) RS pretreated by baseline correction with normalization (RS-BA-NOR). Note: RS, Raman spectrum. BA, baseline. NOR-normalization. The mean spectrum and corresponding standard deviation of each measurement group are displayed in different colors, and the standard deviation is indicated by the shading accompanying each mean spectrum line.

Figure 6a shows similar morphological features of codfish samples within the examined Raman shift region, but the magnitude of spectra intensity fluctuates with the identity difference of the cod. There are significant differences in RS of eight species of codfish, especially at the peaks and valleys of 1007 cm^−1^, 1262 cm^−1^, 1278 cm^−1^, 1319 cm^−1^, 1459 cm^−1^ and 1662 cm^−1^, indicating that Raman intensity of eight species of codfish can also be classified by using RS. All these particular spectral bands reflected by the measured spectra allow the detection and classification of codfish identity and origin.

To specify, the characteristics of the RS of cod, including near 1269, 1306, 1443, 1470, 1660, and 1750 cm^−1^, could be assigned to the C=O stretching vibration, CH_2_ scissoring vibration, C-C stretching vibration, CH_2_ twisting vibration, and CH in plane deformation vibration observed, and they are attributed to fat [40]. The 1004 cm^−1^ is linked to phenylalanine ring stretching vibration, 1230–1350 cm^−1^ linked to amide III region, 1600–1700 cm^−1^ linked to amide I region, and it is attributed to protein. The amide III region (1230–1350 cm^−1^) is a conformationally sensitive band region. This band region can provide vibration information on the main conformation of the polypeptide chain, including C-N stretching vibration, N-H in-plane bending vibration, C-C stretching vibration and C = O in-plane bending vibration [41]. The amide I region (1600–1700 cm^−1^) provides more information for the resolution of the protein secondary structure. This band region not only contains the information on C = O stretching vibration, but also provides information on C-N stretching vibration, C-C-N bending vibration and N-H in-plane bending vibration in the polypeptide group [42]. In addition, the water broad Raman band between 3100 and 3500 cm^−1^ which is attributable to O-H stretching motions [40]. The spectral interference associated with hydrogen bonding is greatly reduced in RS compared to NIRS. By comparing the characteristic peak positions and relative peak intensities of codfish samples in the above important RS bands, the specific effects of identity on cod RS can be explained. 

#### 3.2.2. Selection of Pretreatment Methods for RS

To improve the accuracy and robustness of the spectrum, several RS pretreatment methods were utilized. PLS-DA calibration models were built to correlate the corrected data across full wavelengths with codfish labels, of which the calibration model based on BA with NOR preprocessing yielded acceptable results, with SEC, SPC, ACC all around 88% for the calibration set, and SEP of 76%, SPP of 89%, and ACP of 83% for the prediction set. In this research, the BA combined with a NOR transform correction algorithm was selected for the preprocessing of RS information (Figure 6b). In terms of function, BA was mainly used to eliminate the effects of solid particle size, surface scattering and light range variations on the diffuse reflection spectrum [43]. NOR was mainly used to calibrate spectral changes caused by small optical path differences [44].

#### 3.2.3. Extraction of Effective Wavenumbers

**Figure 7:**
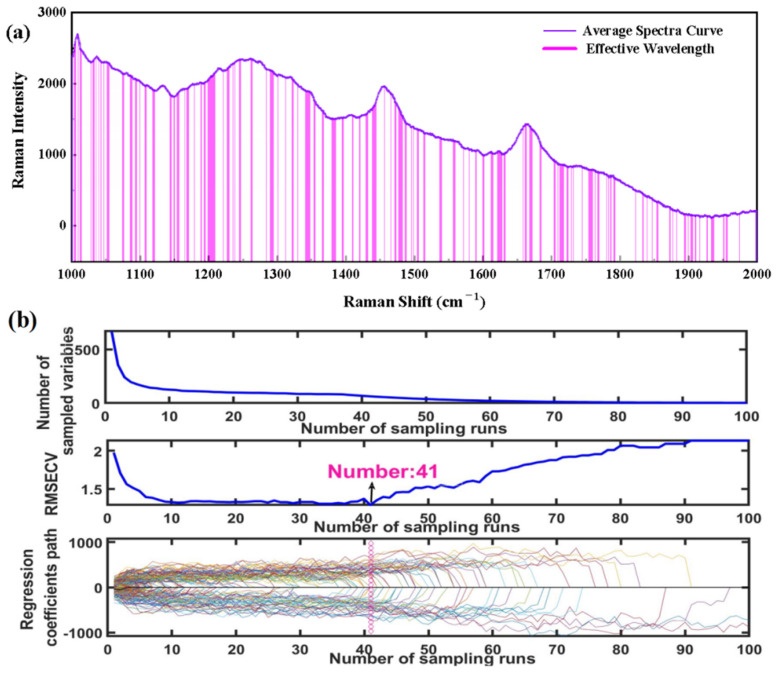
Selection of feature wavenumbers based on (**a**) IRIV-Raman, (**b**) CARS-Raman.

For the RS, the same characteristic wavenumbers selection methods to simplify the model were adopted. The selected feature wavenumbers were assessed and compared to verify the validity of the selected wavelengths and rapid determination of codfish identity. Figure 7a,b demonstrates the selected wavenumbers of IRIV and CARS algorithms, respectively. Table 2 shows the PLS-DA model established by selecting characteristics wavenumbers by the IRIV, CARS and SPA algorithms. As shown in Table 2, 134 characteristics wavebands obtained by the IRIV method, 64 characteristics wavebands selected by the CARSA method, and nine characteristics wavebands obtained by the IRIV method are used for cod identity prediction. In terms of the SPA method, the results showed that 9 (1009, 1005, 1339, 1444, 1661, 1012, 1247, 1450, 1740 cm^−1^) optimal wavenumbers were identified for RS.

#### 3.2.4. Modeling Based on Optimal Wavenumbers

In terms of the results, the relatively more accurate model for assessing codfish identity was established using the PLS-DA model based on key variables extracted by the IRIV method (accounting for 20.03% of 669 total wavebands); the SEC = 88.29%, SECV = 76.40%, SPC = 91.36%, SPCV = 91.10%, ACC = 90.42%, ACCV = 84.35%). Although the number of wavelengths was greatly decreased by the SPA method, the spectral data in the calibration set were reduced to small matrixes as 240 × 9 (samples × variables); the model was overfitting. These results indicated that the key wavebands identified by IRIV were informative and relevant to the identity of codfish. The number of variables was significantly reduced (by 79.97%) by IRIV, indicating further that the IRIV algorithm was effective in eliminating the redundant information. To further verify the credibility of the simplified IRIV-PLS-DA model, the measured and predicted values of the 80 samples in the prediction set were compared, and the SEP, SPP, ACP were obtained, with 65.78%, 89.41%, 77.86%, respectively. It is worth noting that, compared with the full RS wavelength models, the prediction accuracy of the simplified models generally declined. The reason may be that these feature selection algorithms have lost some key waveband information. Therefore, in the subsequent data fusion pipeline, the results of the NIRS characteristic band classification model and the RS full band classification model were fused by the Bayesian method. 

From the classification results, the accuracy of the NIRS modeling classification is higher than that of RS. The reason may be that the RS equipment used in this study is the microscope dispersion spectrometer. However, in the sample detection, the laser frequency of the RS is the key. We only use 532 nm, and fluorescence affects the spectral signal of the sample to a certain extent. Using lasers at 785 nm and 830 nm might overcome the fluorescence problem; most food samples have fluorescence, which may mask other peaks and create problems in the identification. This may be the reason for the low performance of the RS classification model.

### 3.3. Analysis of Bayesian Fusion Data Results

The process of obtaining the fusion classification results started from the earlier experiments, where the node of predicted values of the NIRS and the RS PLS-DA model were obtained, respectively. According to the node value, the probability of each cod sample belonging to a different identity was calculated. Following recalculation with the new probability value, the new post-fusion discriminant result was obtained. In the process of analysis, we noticed that as long as a single spectral technique can distinguish correctly, accurate results can still be obtained with the Bayes information fusion. The Bayes information fusion belongs to decision level fusion, as mentioned earlier. However, for the samples that are wrongly classified by both spectral techniques, the fusion method was unable to correctly identify them. For such samples, the causes need to be found from the source, such as whether the spectrum was acquired correctly, or other reasons.

**Figure 8:**
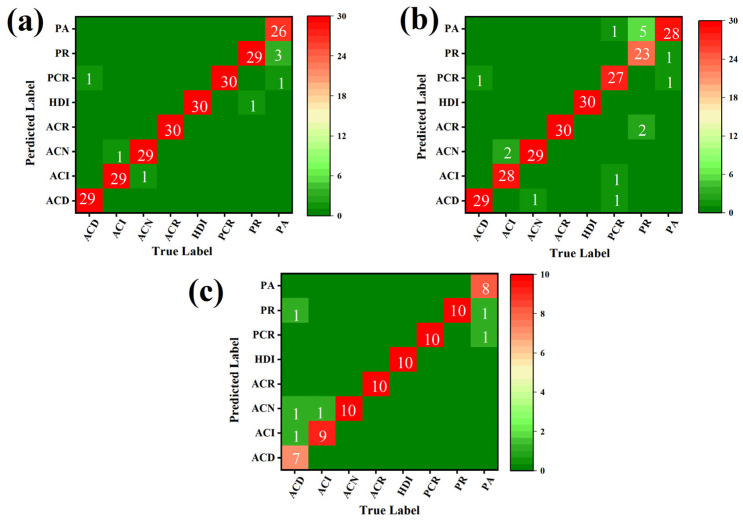
Discrimination result (confusion matrix) of NIR and Raman spectrum Bayesian information fusion model for: (**a**) calibration set, (**b**) cross-validation set, (**c**) prediction set.

Figure 8 shows the codfish spectrum PLS-DA Bayesian data fusion confusion matrix of calibration, CV and prediction sets. The discriminant results of the fused model are shown in Table 3. The results of data fusion showed that the sensitivity, specificity, and accuracy of prediction set reached 92.50%, 98.93% and 98.12%, respectively, which were significantly improved compared with single NIRS (85%, 96.25%, 90.63%, respectively) or RS (76.25%, 89.10%, 82.68%, respectively) classification metrics. Compared with NIRS-RS-D (81.25%, 96.59%, 88.93%, respectively) and NIRS-RS-F (85%, 96.79%, 90.89%, respectively), the model performance of NIRS-RS-B discrimination was also improved. Without doubt, after Bayesian fusion, the classification model of integral differential rate performed better than a single spectral data classification model.

## 4. Conclusions

The current codfish identification management and traceability system is based on the industry integrity of enterprises, and the producers ensure the authenticity of the identification content. However, more and more adulteration incidents prove that it is not enough to rely solely on enterprises or industries to perform their due diligence. In this research, a novel Bayesian information fusion model was presented merging the NIRS and RS to improve the accuracy of cod identity prediction. In some cases, either NIRS or RS could be a valid pre-screening technique, to test codfish identity when speed and cost of analysis matter. However, as validated by an external prediction set as well as internally, the Bayesian fusion model outperforms both techniques in all the metrics studied. Experimental results also show that the NIRS-RS Bayesian fusion approach produces superior results in comparison with those obtained by the NIRS-RS-D or NIRS-RS-F. The NIRS-RS-B approach reliably classified codfish with over 92% sensitivity, 98% specificity, and 98% accuracy. Hence, the Bayesian fusion of information-based discrimination methods and discrimination models provides a new strategy and possible approach to develop novel methodologies with high efficiency and low cost for identification of codfish.

Meanwhile, the Bayesian fusion algorithm is a relatively new approach to merge the data from different sources (spectrum, chromatography, mass spectrometry et al.) at the decision level to improve the prediction performance. Further work should also be undertaken to clarify whether spectral classification is affected by seasonal variations, treatment methods and different storage conditions, to broaden the application of classification results. These important findings can help improve the fight against commercial fraud, extending the possibility to authenticate fish identity also in, e.g., processed products. In the future, we will focus on how to further improve the computational speed of the algorithm and apply our Bayesian model approach to other image fusion and signal processing fields.

## Figures and Tables

**Table 1:** Performance comparison of PLS-DA models with different pretreatment methods for codfish NIR/Raman spectrum in the full waveband range (variables).

Pretreatment Method	Number of Variables	LVs	Calibration Set			Cross-Validation Set			Prediction Set		
			SEC	SPC	ACC	SECV	SPCV	ACCV	SEP	SPP	ACP
NIR-None	2593	6	86.26	85.88	86.07	85.00	85.54	85.27	85.00	85.38	85.18
NIR-NOR	2593	6	86.68	85.83	86.25	84.59	85.23	84.91	85.00	85.00	85.00
NIR-MC	2593	6	85.84	88.33	87.08	84.16	87.78	85.98	88.75	88.21	88.48
NIR-MSC	2593	7	87.51	89.23	88.36	87.10	88.91	88.01	83.75	88.21	85.98
NIR-SNV	2593	7	87.51	89.29	88.39	87.10	88.91	88.01	83.75	88.21	85.98
NIR-FD	2593	6	90.81	85.88	88.36	74.18	86.83	80.51	68.75	85.88	77.32
NIR-BA	2593	5	87.19	85.25	84.55	85.20	85.60	83.15	84.53	84.96	83.66
NIR-SNV with MC	2593	7	89.81	92.19	89.64	89.34	91.57	89.08	89.53	90.84	87.95
Raman-None	669	5	78.39	73.44	76.99	72.74	72.94	74.23	66.25	74.57	71.79
Raman-NOR	669	7	82.13	86.49	85.00	73.17	86.16	81.13	60.47	87.61	75.80
Raman-SG	669	8	80.40	85.30	82.86	75.00	84.34	79.67	68.75	83.58	76.16
Raman-SNV	669	7	77.93	82.98	80.45	67.93	82.26	75.09	53.75	81.43	67.59
Raman-BA	669	6	83.50	84.70	83.01	77.77	84.44	79.97	68.91	84.85	76.43
Raman-SNV with NOR	669	7	75.58	86.02	80.45	64.33	85.22	75.09	47.97	84.64	67.59
Raman-BA with NOR	669	6	88.76	87.19	87.98	78.33	88.03	83.18	76.25	89.10	82.68
Raman-SG with NOR	669	6	77.51	82.09	79.79	74.99	81.29	78.15	60.00	83.40	71.70

Note: LV = Latent Variable. MC: Mean centered. SEC: Sensitivity of Calibration set. SPC: Specificity of Calibration set. ACC: Accuracy of Calibration set. SECV: Sensitivity of Cross validation set. SPCV: Specificity of Cross validation set. ACCV: Accuracy of Calibration set. SEP: Sensitivity of prediction set. SPP: Specificity of prediction set. ACP: Accuracy of prediction set. BA: Baseline. NOR: Normalize. SG: Savitzky-Golay smoothing. All numbers are expressed as percentages.

**Table 2:** Performance (%) of PLS-DA models based on preprocessing spectra in the selected feature wavenumbers (i.e., variables).

Modelling Profile	Variable Amounts	LVs	Calibration Set			Cross-Validation Set			Prediction Set		
			SEC	SPC	ACC	SECV	SPCV	ACCV	SEP	SPP	ACP
SNV-MC-CARS-NIRS	93	13	95.85	97.24	96.55	91.68	96.08	93.87	83.75	96.55	89.64
SNV-MC-IRIV-NIRS	83	17	98.34	97.96	98.15	91.26	96.3	93.78	85.00	96.25	90.63
SNV-MC-SPA-NIRS	9	7	84.20	89.25	86.34	89.35	89.10	86.01	86.88	87.90	86.25
BA-NOR-CARS-RS	64	8	88.75	88.68	88.72	73.35	87.55	80.45	65.00	86.78	75.89
BA-NOR-IRIV-RS	134	8	88.29	91.36	90.42	76.40	91.10	84.35	65.78	89.41	77.86
BA-NOR-SPA-RS	9	9	81.03	82.45	81.43	74.10	80.13	76.94	60.16	80.10	70.27

Note: All numbers are expressed as percentages. LVs, Latent Variables. SNV-MC, standard normal variation with mean centering. BA-NOR, baseline correction with normalization (spectrum preprocessing algorithm). CARS, competitive adaptive reweighted sampling. IRIV, iteratively retaining informative variables. SPA, successive projections algorithm (spectrum feature selection algorithm). NIRS, near infrared spectrum. RS, Raman spectrum. ACC, accuracy of calibration. ACCV, accuracy of cross-validation. ACP, accuracy of prediction. SEC, sensitivity of calibration. SECV, sensitivity of cross-validation. SEP, sensitivity of prediction. SPC, specificity of calibration. SPCV, specificity of cross-validation. SPP, specificity of prediction. All numbers are expressed as percentages.

**Table 3:** Comparison of data fusion performance (%) at different modes for codfish identification.

Data Fusion Mode	Calibration Set			Cross-Validation Set			Prediction Set		
	SEC	SPC	ACC	SECV	SPCV	ACCV	SEP	SPP	ACP
Bayesian information fusion	96.67	99.40	99.06	93.33	99.05	98.33	92.50	98.93	98.12
Feature layer fusion	98.76	98.44	98.60	93.78	97.13	95.45	81.25	96.59	88.93
Data layer fusion	98.76	98.20	98.48	92.51	97.28	94.88	85.00	96.79	90.89

Note: All numbers are expressed as percentages. ACC, accuracy of calibration. ACCV, accuracy of cross-validation. ACP, accuracy of prediction. SEC, sensitivity of calibration. SECV, sensitivity of cross-validation. SEP, sensitivity of prediction. SPC, specificity of calibration. SPCV, specificity of cross-validation. SPP, specificity of prediction. As complimentary methods, NIRS and RS provide complimentary chemical and structural information of the cod samples, which, as demonstrated, has the potential to better illustrate the differences of different cod species and origins. This method can get the correct discriminant result even with samples that were misclassified using each spectral method separately after the application of the Bayes probability formula. In other words, the Bayesian fusion classification model improved the accuracy of classification beyond what a single spectral method classification had achieved. All these results indicate that the models could be suitable for the prediction of codfish identity.

## Data Availability

The data used to support the findings of this study can be made available by the corresponding author upon request.

## Abbreviations

ACC, accuracy of calibration. ACCV, accuracy of cross-validation. ACP, accuracy of prediction. BA, baseline correction. CARS, competitive adaptive reweighted sampling. FD, first derivative. IRIV, iteratively retaining informative variables. MC, mean centering. MSC, multiplicative scatter correction. NIRS, near infrared spectrum. NIRS-RS, NIRS plus RS. NIRS-RS-B, NIRS plus RS fused on decision layer using Bayesian algorithm. NIRS-RS-D, NIRS plus RS fused on data layer. NIRS-RS-F, NIRS plus RS fused on feature layer. NOR, normalization. PLS-DA, partial least squares discriminant analysis. RS, Raman spectrum. SEC, sensitivity of calibration. SECV, sensitivity of cross-validation. SEP, sensitivity of prediction. SG, Savitzky-Golay smoothing. SNV, standard normal variation. SPA, successive projections algorithm. SPC, specificity of calibration. SPCV, specificity of cross-validation. SPP, specificity of prediction.
